# Immunogenicity of a Recombinant Multi-Epitope Vaccine Incorporating GRA14, SAG1, and GRA1 Antigens of *Toxoplasma gondii* in BALB/c Mice

**DOI:** 10.3390/vaccines14060545

**Published:** 2026-06-20

**Authors:** Abdulrahman M. Sheikh, Wong Weng Kin, Robaiza Zakaria, Ahmad A. Alshehri, Mohammed Dauda Goni, Abdulrazzag Abdulaziz Othman, Zakeya Al Rasbi, Zeehaida Mohamed, Khalid Hajissa

**Affiliations:** 1 Department of Medical Microbiology & Parasitology, School of Medical Sciences, Universiti Sains Malaysia, Kubang Kerian 16150, Kelantan, Malaysia; abdulrahmanbilaal@gmail.com (A.M.S.); robaiza@usm.my (R.Z.); 2 School of Health Sciences, Universiti Sains Malaysia, Kubang Kerian 16150, Kelantan, Malaysia; kinnywong84@gmail.com; 3 Department of Clinical Laboratory Sciences, College of Applied Medical Sciences, Najran University, Najran P.O. Box 1988, Saudi Arabia; aaalshehri@nu.edu.sa; 4 Faculty of Veterinary Medicine, Universiti Malaysia Kelantan, Pengkalan Chepa, Kota Baru 16100, Kelantan, Malaysia; dauda.g@umk.edu.my; 5 Department of Clinical Laboratory Sciences, College of Applied Medical Sciences, Shaqra University, Riyadh 11961, Saudi Arabia; aothman@su.edu.sa; 6 Department of Medical Microbiology and Immunology, College of Medicine and Health Sciences, United Arab Emirates University, Al Ain P.O. Box 15551, United Arab Emirates; rasbi@uaeu.ac.ae; 7 Medical Microbiology Laboratory, Hospital Universiti Sains Malaysia, Kubang Kerian 16150, Kelantan, Malaysia; 8 Department of Biology, Faculty of Science and Technology, Omdurman Islamic University, Omdurman P.O. Box 382, Sudan

**Keywords:** *Toxoplasma gondii*, immunoinformatics, multi-epitope, vaccine, immunogenicity

## Abstract

Background: The high incidence and severe health threat of *Toxoplasma gondii* (*T. gondii*) infection, particularly in immunocompromised patients, underscore the urgent need for the development of a safe and effective vaccine. The aim of this study was to develop a novel multi-epitope vaccine (USM.TOXOII) incorporating the *T. gondii* GRA14, SAG1, and GRA1 antigens, and to assess its immunogenicity in BALB/c mice. Methods: Using bioinformatics approach, the USM.TOXOII was designed and evaluated. The encoding gene (471 bp) was then constructed and cloned into the pET-30a (+) plasmid before being transformed into *E. coli* expression system. The recombinant USM.TOXOII protein was subsequently expressed and purified. Finally, an animal study was performed to assess the vaccine’s immunogenicity. Results: The USM.TOXOII protein (17.27 kDa) was soluble and contained a His tag protein. Immunization of BALB/c mice with USM.TOXOII significantly elevated serum levels of total IgG, IgG1, and IgG2a (*p* < 0.05). Cytokine analysis revealed a significant increase in IFN-γ production, whereas IL-4 levels remained unchanged, suggesting a Th1-biased immune response. Conclusions: Collectively, these findings indicate that USM.TOXOII possesses immunogenic potential and is capable of inducing both humoral and cellular immune responses in BALB/c mice. Future challenge studies with live *T. gondii* tachyzoites are warranted to evaluate its protective efficacy in vivo.

## 1. Introduction

*Toxoplasma gondii* is an obligate intracellular protozoan parasite that causes toxoplasmosis, a disease with significant medical, veterinary, and economic importance [1]. As of 2020, at least one billion people worldwide were reported to have been infected with *T. gondii* [2,3]. Global statistics from recent systematic reviews and meta-analyses indicate a substantial burden of *T. gondii* infection; however, seroprevalence rates vary considerably by geography and population. Globally, about 36.6% of pregnant women are seropositive, with some regional studies indicating even higher rates, such as 42.89% among pregnant women in Africa [4,5]. In broader human populations, the prevalence ranges from 32.3% in Spain to as high as 60.06% in Indonesia [6,7]. As an opportunistic parasite, the *T. gondii* infection in immunocompromised individuals or pregnant women may result in life-threatening complications [8]. The disease also leads to economic losses in livestock, particularly sheep and goats, through abortion and/or neonatal loss [9]. Accordingly, effective preventive measures or therapeutic approaches are essential. Nonetheless, current treatments are limited in eliminating chronic infection. Therefore, the development of an effective vaccine against *T. gondii* is crucial.

Although the development of a licensed vaccine against human toxoplasmosis remains an ongoing challenge, considerable progress has been made in recent years and attempts to produce such vaccines have advanced remarkably [10]. In this context, multi-epitope vaccines which contain multiple epitopes or antigenic regions from a pathogen’s proteins have been thoroughly studied because of their simplicity, safety and stability. Such vaccines have the potential to boost immune responses while also reducing the likelihood of immunological escape and pathogen resistance development, making them extremely promising for the development of effective and unique vaccination solutions [11,12].

The recent validation of immunoinformatic approaches for developing a novel and effective epitope-based vaccine against *T. gondii* has shown promising potential, with numerous studies demonstrating that such vaccines can elicit strong protective responses by stimulating both cellular and humoral immunity. In this context, several in silico vaccine design attempts have successfully identified and combined multiple epitopes from the key antigens of *T. gondii*’s invasion machinery. This includes surface antigens (SAGs), rhoptry proteins (ROPs), microneme proteins (MICs), dense granule antigens (GRAs), and various accessory molecules, all of which have been widely recognized as promising targets for vaccine development against toxoplasmosis. For instance, our group has developed a multi-epitope vaccine utilizing ROP2, MIC3, and GRA7, which demonstrated strong in silico immunogenicity and structural stability [13]. A second construct, based on SAG1, GRA2, and GRA7 antigens, was further validated in vivo using BALB/c mice, where it significantly induced a mixed Th1/Th2 immune response [14]. Similarly, Moghadamizad et al. developed a multi-epitope vaccine based on ROP5, ROP7, and SAG1. This vaccine elicited Th1-dominant immune responses, significantly reduced the cerebral cyst load, and provided partial protection against chronic toxoplasmosis in a murine model [15]. In contrast, a computational framework was also utilized to construct epitope-based DNA vaccines, which successfully induced strong humoral and cellular immunity in BALB/c mice, resulting in a reduced brain cyst burden and enhanced survival rates [16,17].

Furthermore, similar approaches have also been successfully applied to *Plasmodium falciparum*, *Leishmania donovani*, and *Trypanosoma cruzi*, where immunoinformatics has significantly accelerated the identification of immunogenic candidates [18,19,20]. Despite the advantages of utilizing immunoinformatics approaches in vaccine design, which enable the rapid identification of promising targets, a key limitation is the reliance on predictive algorithms that may not account for post-translational modifications, host–parasite interactions in vivo, or genetic diversity across strains. Moreover, most of these studies remain at the computational level, with limited validation through experimental or clinical studies.

In this study, a robust immunoinformatics approach was first used to develop a novel multi-epitope vaccine expressing three *T. gondii* antigens (GRA14, SAG1, and GRA1). The candidate antigens were chosen due to their immunogenicity and diagnostic value. GRA14 moves to the intravacuolar system and the parasitophorous vacuole membrane after being secreted into the vacuole [21], making it a viable target for immunological treatments since it is extremely common among *T. gondii* strains and is produced during both the acute and chronic phases of infection [22,23].

The SAG1 constitutes approximately 5% of the tachyzoite antigen and is one of the major surface antigens of *T. gondii* [24]. The primary role of SAG1 is to facilitate host cell invasion by parasites. Co-immunization with DNA vaccines co-expressing SABP1 and SAG1 has been shown to significantly enhance resistance to acute T. gondii infection in BALB/c mice, as evidenced by elevated parasite-specific IgG levels, increased IFN-γ production, and improved survival rates [25]. Furthermore, immunization of mice with full-length recombinant SAG1 protein significantly reduced mortality following lethal T. gondii challenge, accompanied by robust IgG1 and IgG2a antibody responses and upregulated IFN-γ production [26]. These results consistently confirm SAG1 as a high-priority immunogenic component for multi-epitope vaccine design. GRA1, however, has been recognized as a major antigen by the host immune system, being capable of activating both innate and adaptive responses. Infections have been shown to lead to the presence of GRA1-specific antibodies in the sera from infected individuals [27]. In addition, it is known that GRA1 is a highly immunogenic antigen that stimulates strong humoral and cellular immune responses [28]. Accordingly, *T. gondii* GRA14, SAG1, and GRA1 were chosen as promising candidate antigens for developing a vaccine against this parasite. The vaccine was designed and evaluated using reverse vaccinology and immunoinformatics approaches. Furthermore, the effectiveness and immunogenicity of the developed vaccine were assessed through mouse experiments.

## 2. Materials and Methods

### 2.1. Protein Sequence Retrieval

The protein sequences of *T. gondii* GRA14 (accession number AXG50779.1), SAG1 (accession number AAO61460.1), and GRA1 (accession number KAF4642298.1) antigens were obtained from the NCBI portal (https://www.ncbi.nlm.nih.gov/genbank/, accessed on 25 March 2022) in FASTA format.

### 2.2. Prediction of Linear B-Lymphocyte (LBL) Epitopes

The potential LBL epitopes in the sequences of selected proteins were predicted using the ABCpred server (https://webs.iiitd.edu.in/raghava/abcpred/, accessed on 25 March 2022) with default settings. Using machine learning techniques, this prediction tool distinguishes between experimental and uncontrolled B-cell receptors. A 16 amino acid receptor length, overlap filter usage, and 75% specificity were among the criteria used. To ascertain the human homology of the selected epitopes, the BLASTp option at NCBI (https://blast.ncbi.nlm.nih.gov/, accessed on 30 March 2022) was used.

### 2.3. Prediction of Cytotoxic T-Lymphocyte (CTL) Epitopes

The CTL epitopes of the three chosen *T. gondii* antigens were identified utilizing the NetCTL v1.2 online server (https://services.healthtech.dtu.dk/services/NetCTL-1.2/; threshold value = 0.75, accessed on 02 April 2022) [29]. The antigenicity of the expected epitope targets were further evaluated by VaxiJen v2.0, at (https://www.ddg-pharmfac.net/vaxijen/VaxiJen/VaxiJen.html, accessed on 03 April 2022) [30].

ToxinPred (http://crdd.osdd.net/raghava/toxinpred/, accessed on 03 April 2022) was then utilized to evaluate the toxicities, while AllergenFP V1.0 (https://ddg-pharmfac.net/AllergenFP/, accessed on 03 April 2022) was utilized to estimate the allergenicity [3], and Epitope conservancy was analyzed using the IEDB Epitope Conservancy Analysis tool (https://tools.iedb.org/conservancy/, accessed on 03 April 2022). Server’s standard parameters were applied to the aforementioned predictions.

### 2.4. Prediction of Helper T-Lymphocyte (HTL) Epitope

The helper T-lymphocyte (HTL) epitopes that bind to MHC class II molecules were predicted as 15-mer epitopes using the IEDB server (http://tools.iedb.org/mhcii/, accessed on 6 April 2022). Here, the Human/HLA-DR (species/locus) and 7-allele human leukocyte antigen (HLA) were used as references for predicting the HTL epitopes. Epitopes were selected using a 5% average percentile and the CONSENSUS 2.22 technique. Toxicity and allergenicity were predicted as well using their respective portals. Moreover, the predicted epitopes were then evaluated for their potential to stimulate IL-10, IL-4, and IFN-γ using the default parameters in IL10pred, IL4pred, and IFNepitope online servers.

### 2.5. Construction of the USM.TOXOII Vaccine and Evaluation of Its Properties

To design the USM.TOXOII vaccine construct, the selected CTL, HTL, and LBL epitopes were combined using appropriate linkers. The GPGPG, AAY and KK linkers were used because of their potential role in protein folding, flexibility, and epitope separation and stability. The ProtParam web platform (http://web.expasy.org/protparam/, accessed on 20 April 2022) was used to estimate the physiochemical characteristics and understand the essential nature of the final vaccine construct. The physiochemical data include the number of amino acids, molecular size, hypothetical isoelectric point, isotope and mineral layout, extinction coefficients, formula, aliphatic index, projected lifespan, instability index, and the overall mean of a hydrated (GRAVY). The VaxiJen 2.0 platform and SOLPro (https://scratch.proteomics.ics.uci.edu/, accessed on 20 April 2022) were used to forecast the vaccine’s antigenicity and solubility, respectively.

### 2.6. Secondary and Tertiary Structure Prediction and Validation

The secondary structure of the vaccine was predicted using the PSIPRED (http://bioinf.cs.ucl.ac.uk/psipred, accessed on 20 April 2022). The tertiary structure was predicted using the trRosetta server (https://yanglab.qd.sdu.edu.cn/trRosetta/, accessed on 20 April 2022), and the best model was selected and subsequently refined using GalaxyRefine (https://galaxy.seoklab.org, accessed on 25 April 2022). The quality of the refined model was evaluated using ProSA-web (https://prosa.services.came.sbg.ac.at/prosa.php, accessed on 27 April 2022) to determine the Z-score, and ERRAT (https://saves.mbi.ucla.edu/, accessed on 27 April 2022) to assess the overall structural reliability.

### 2.7. Molecular Docking of USM.TOXOII Vaccine with TLR-4 Receptor

Interaction of the USM.TOXOII vaccine candidate with TLR-4 can improve its immunization efficacy. Accordingly, protein–protein docking has been employed to determine the binding affinity of the USM.TOXOII with TLR-4. The ClusPro v2.0 server (https://cluspro.org/, accessed on 30 April 2022) was used to dock the USM.TOXOII vaccine’s construct with the TLR-4. In the docking approach, the 3D structures of TLR-4 (4G8A) were obtained from the RCSB protein data bank and used as the receptor, with USM.TOXOII serving as the ligand. The PyMOL molecular Graphic system version 2.0 was further used to model the cluster platform’s output.

### 2.8. Synthesis and Cloning of USM.TOXOII Gene

The amino acid sequence of the designed USM.TOXOII vaccine was reverse-transcribed into a DNA sequence using the Expasy reverse translation tool (Data S1), which was then codon-optimized using the Java Codon Adaptation Tool (JCat, http://www.jcat.de/, accessed on 10 May 2022) for effective expression in the *E. coli* system. The codon-optimized gene was then synthesized by Integrated DNA Technologies, Inc. (Singapore). Subsequently, the synthesized USM.TOXOII gene was cloned into the *NheI* and *HindIII* restriction enzyme sites of the prokaryotic expression vector pET30a (+) plasmid.

### 2.9. Expression and Purification of the USM.TOXOII Recombinant Protein

*Escherichia coli* strain BL21 (DE3) pLysS competent cells were used for the expression of USM.TOXOII recombinant antigen. First, the pET30a.USM.TOXOII recombinant plasmid was transformed into BL21 (DE3) pLyS competent cells and cultured in LB broth medium containing 50 µg/mL kanamycin at 37 °C overnight while shaking vigorously (225 rpm). The following day, 1 mL of the overnight cultures was added to 100 mL of the LB medium and grown until the OD at 600 nm reached 0.4–0.6. The expression of the USM.TOXOII recombinant protein was induced using isopropyl ß-D-1-thiogalactopyranoside (IPTG) at a final concentration of 1 mM. The expressed USM.TOXOII His-tagged protein was verified by standard Western blotting analysis with anti-histidine antibodies or *T. gondii*-positive human sera, prior to being purified using a Ni-NTA spin column (Qiagen, Hilden, Germany) as per the manufacturer’s instructions. The human sera were collected from the remaining serum samples requested for routine *T. gondii* serological investigation at the serology laboratory at HUSM, following approval from the Human Research Ethics Committee of Universiti Sains Malaysia (HREC) (Approval number USM/JEPeM/20030168).

### 2.10. Mice Immunizations

The study protocol was approved by the Animal Ethics Committee of the Universiti Sains Malaysia (USM/IACUC/2O22/(136)(1211)). The animal experiment was carried out in accordance with relevant guidelines and regulations and was reported in accordance with ARRIVE guidelines. A total of 28 male BALB/c mice, aged 8 to 10 weeks, were obtained from the Animal Research and Service Center (ARASC) and were randomly divided into four groups, with seven mice per group (*n* = 7). Groups B and D were vaccinated with three injections of 1 µg of USM.TOXOII per mouse in a volume of 100 µL, either intraperitoneally (B) or intradermally (D). The two remaining groups (A and C) were regarded as control groups and received 100 µL PBS injections in a similar manner via intraperitoneal (C) and intradermal injections (D). Freund’s complete adjuvant was used to emulsify the USM.TOXOII or PBS in the prime dosage (on day 0), and Freund’s incomplete adjuvant was used during the subsequent booster doses on days 14 and 28.

### 2.11. Quantification of USM.TOXOII-Specific IgM, IgG and IgG Subclasses by Enzyme-Linked Immunosorbent Assay (ELISA)

Blood samples were collected from all mice and centrifuged at 6000× *g* for 15 min to obtain sera. Sera were pooled within each experimental group separately prior to ELISA analysis. Subsequently, the collected mouse sera were used to quantify the presence of USM.TOXOII-specific IgM, IgG and IgG subclass antibodies using ELISA. Briefly, 0.05 M carbonate buffer (pH 9.6) was used to dilute the purified USM.TOXOII to 5 µg/mL. Each 96-well microplate was then filled with 100 microliters of this dilution and incubated overnight at 4 °C. Following the incubation, each plate was rinsed three times for 5 min using PBS-T. To eliminate the non-specific binding, 200 µL of the blocking reagent was added to each well, after which the plate was incubated for one hour at 37 °C. Each microtiter plate well was then filled in triplicate with 100 µL of pooled sera from each experimental group separately diluted in blocking buffer (1:200) and incubated for 30 min at 37 °C. The wells were rinsed three times before adding 100 µL of Horseradish peroxidase (HRP)-conjugated anti-mouse IgM, IgG, IgG1, IgG2a, and IgG2b antibodies at a dilution of 1:10,000 in PBS. The plate was then incubated for an additional 30 min at 37 °C. After three additional washing cycles, 100 µL of the substrate was applied and left to stand for 15 min. Finally, 100 µL of 2 M H_2_SO_4_ was added to halt color development, and the density of light was measured using microplate readers at 450 nm.

### 2.12. Splenocyte Proliferation and Cytokine Analysis

Two weeks after the final injection, mice were euthanized by intraperitoneal injection of ketamine overdose (200 mg/kg) and xylazine (5 mg/kg) in accordance with the approved animal ethics protocol. Spleens were surgically removed from all groups, and splenocytes were isolated by mechanical disruption. Splenocytes from USM.TOXOII-immunized mice or control groups were resuspended at 5 × 10^6^ cells/mL in RPMI 1640 supplemented with 10% FBS. Subsequently, 100 µL of the cell suspension and 100 µL of complete RPMI 1640 medium containing 10 μg/mL of purified USM.TOXOII protein was added to 96-well culture plates. In parallel, cells were cultured with PBS as a negative control or Concanavalin A as a positive control. All splenocyte cultures were carried out in triplicate. The plates were then incubated for 24 and 72 h for IL-4 and IFN-γ, respectively, at 37 °C in a humidified environment with 5% CO_2_. Following the incubation period, 100 µL of supernatant per well was harvested and stored at −80 °C for subsequent cytokine-detection assays. The mouse IFN-γ and IL-4 levels were measured using Elabscience^®^ ELISA kits (Elabscience^®^, Houston, TX, USA) following the manufacturer’s instructions.

### 2.13. Statistical Analysis

Statistical analysis was performed using IBM SPSS Statistics (version 26). Data were expressed as mean ± SD, and *p* < 0.05 was considered statistically significant. Differences between groups were analyzed using an independent samples *t*-test. Repeated measures ANOVA followed by a Bonferroni post hoc test was used to assess changes over time. ELISA and cytokine data were derived from pooled group sera analyzed in triplicate across three independent biological experiments; the reported mean ± SD reflects inter-assay and intra-assay variability.

## 3. Results

### 3.1. Sequence Retrieval and Antigenicity Assessment

The amino acid sequences (in FASTA format) of three typical *T. gondii* proteins (GRA14 (408 aa), SAG1 (319 aa), and GRA1 (190 aa)) were obtained from NCBI. The Vaxijen v2.0 server identified all of them as antigenic proteins, with antigenicity scores of 0.5337, 0.7719, and 0.4815 for GRA 14, SAG1, and GRA1 (Table S1).

### 3.2. Identification of Potential LBL Epitopes

A total of 10 LBL epitopes for GRA14, 9 for SAG1 and 5 for GRA1 were initially identified using the ABCpred server, each of which was 16 amino acids in length. Twelve of them were considered appropriate after being further evaluated for antigenicity, allergenicity, and toxicity. To standardize the final construct with the number of selected CTL and HTL epitopes, only the top three LBL epitopes with the highest cumulative scores were selected for inclusion in the final vaccine design (Table S2).

### 3.3. Identification of Potential CTL Epitopes

A total of 83 potential CTL epitopes with cumulative scores higher than 0.75 were identified by the NetCTL v1.2 server, each being nine amino acids long. Alongside comprehensive evaluations of antigenicity, toxicity, allergen potential, and immunogenic properties, only three CTL epitopes met the criteria for being antigenic, non-allergenic, immunogenic, and non-toxic. Consequently, only these three were incorporated into the final vaccine construction (Table S3).

### 3.4. Identification of Potential HTL Epitopes

A total of 258 HTL epitopes, each including 15 amino acids, were predicted by the IEDB server. Following the assessment of their nontoxic, antigenic, non-allergenic properties, the epitopes’ abilities to induce IFNγ, IL-4 and IL-10 were predicted. Among the obtained HTL epitopes, 3 were capable of inducing at least two of the assessed cytokines—IFN-γ and IL-10—while also meeting the requirements for antigenicity, non-toxicity, non-allergenicity, absence of human homology, and 100% conservancy. These epitopes were thus selected for the final vaccine formulation (Table S4).

### 3.5. Multi-Epitope Vaccine Construction

**Figure 1:**
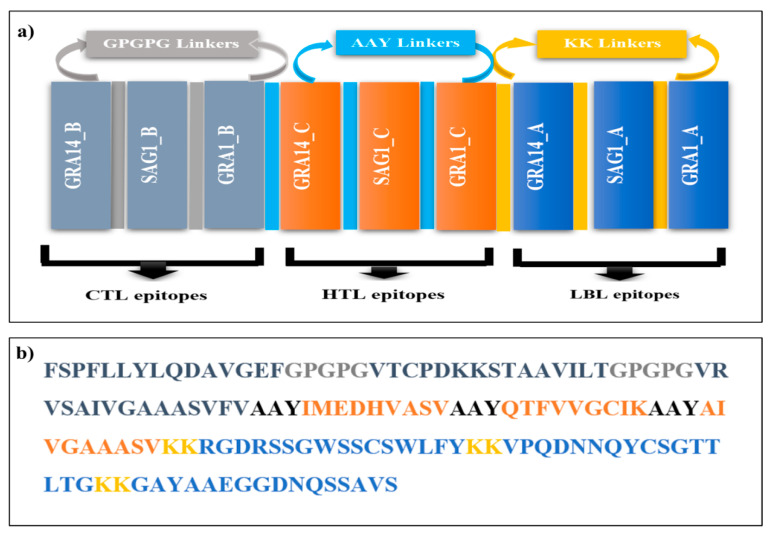
The designed *T. gondii* multi-epitope vaccine. (**a**) Graphical illustration of the USM.TOXOII vaccine construct. (**b**) A one-letter illustration of the principal design of the developed vaccine in amino acid format, CTL epitopes (dark gray) linked by GPGPG linkers (gray), HTL epitopes (orange) linked by AAY linkers (black), and linear B-cell epitopes (blue) linked using KK linkers (yellow).

A final multi-epitope vaccine construct consisting of 145 amino acids was designed using nine LBL, CTL, and HTL epitopes (3 each). These epitopes were linked together using KK, GPGPG, and AAY linkers (Figure 1).

### 3.6. Evaluation of the Physicochemical Properties, Antigenicity, Allergenicity and Solubility of the Constructed Vaccine

The ProtParam portal revealed that the molecular weight of the vaccine candidate was 14.8 kDa, and the theoretical isoelectric point was 8.69, showing its basic nature. The computed instability index of USM.TOXOII (34.85) indicates that the designed vaccine is a stable protein. Moreover, the aliphatic index was 76.76, and the GRAVY (Grand Average of Hydropathicity) value was 0.163, indicating the hydrophobic nature of the USM.TOXOII (Table 1). The antigenicity and solubility of USM.TOXOII were estimated to be 0.7758 (antigenic) and 0.5155555, respectively. Furthermore, the construct demonstrated non-allergenic characteristics.

### 3.7. Secondary Structure Prediction

The analysis of the secondary structure of USM.TOXOII revealed that the designed structure consists of 37.9% α helices, 12.4% β-strands, and 49.7% random coils (Figure S1).

### 3.8. Prediction, Refinement, and Validation of Tertiary Structure

**Figure 2:**
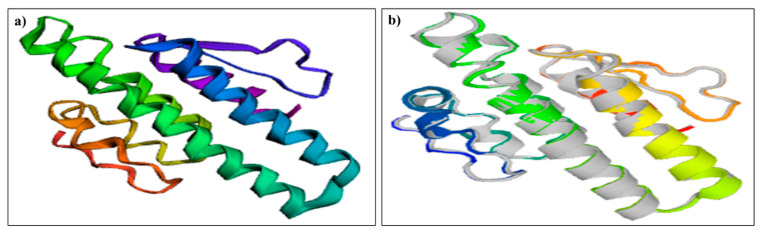
Predicting and refining the tertiary model of the USM.TOXOII vaccine. (**a**) A 3D representation of a developed vaccine. (**b**) The Galaxy-refined structure of the vaccine’s model.

**Figure 3:**
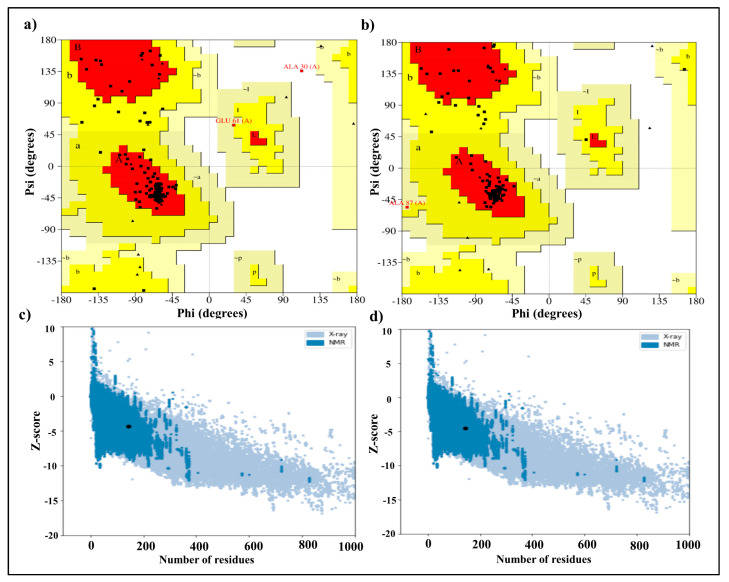
3D model’s validation of the designed construct. (**a**,**b**) The vaccine’s Ramachandran architectural plot and statistics before and after refinement. (**c**,**d**) Z-score of the construct before and after refinement estimated by ProSA Server.

From the trRosetta server, the tertiary structure of the USM.TOXOII vaccine-construct was predicted. Among the five models created by the server, a comparatively higher-ranked model (Figure 2a) with an estimated TM score of 0.241 was considered the best and selected for refinement analysis using the GalaxyRefine server. As a result, five refined models were generated, with model 1 (Figure 2b) being subjected to additional analysis because it demonstrated the lowest RMSD score (0.282), a GDT-HA score of 0.9931, MolProbity score of 2.231, no rotamer scores, and Rama favored score of 94.4. The validation via Ramachandran plot analysis conducted by the PROCHECK server indicated that 90.7% of residues were located in the most preferred region, 6.8% in extra authorized regions, 1.0% in generously authorized regions, and 1.4% in banned regions, as seen in Figure 3. Additionally, ERRAT indicated that the protein achieved a complete quality score of 96.3235, and verification revealed that 57.24% of the amino acids exhibited an average 3D-1D score of at least 0.1. The model also yielded a Z score of −6.57, and the Q-mean tool, which indicates whether the quality of the refined vaccination is likely within the limits of the non-redundant set of PDB structures necessary for proteins of acceptable quality, yielded a Q-mean of −3.58.

### 3.9. Molecular Docking with TLR

Molecular docking analysis was carried out to explore the stability and binding affinity between the USM.TOXOII and TLR-4 receptor using the ClusPro v2.0 server. The designed vaccine showed a docking score of −1039.6 Kcal/mol, indicating a favorable predicted binding energy and suggesting a potential interaction with the TLR-4 receptor. PyMol software (version 2.5) was employed to visualize the 3D docking results of the vaccine’s receptor complex (Figure S2).

### 3.10. Production of USM.TOXOII Multi-Epitope Antigen

**Figure 4:**
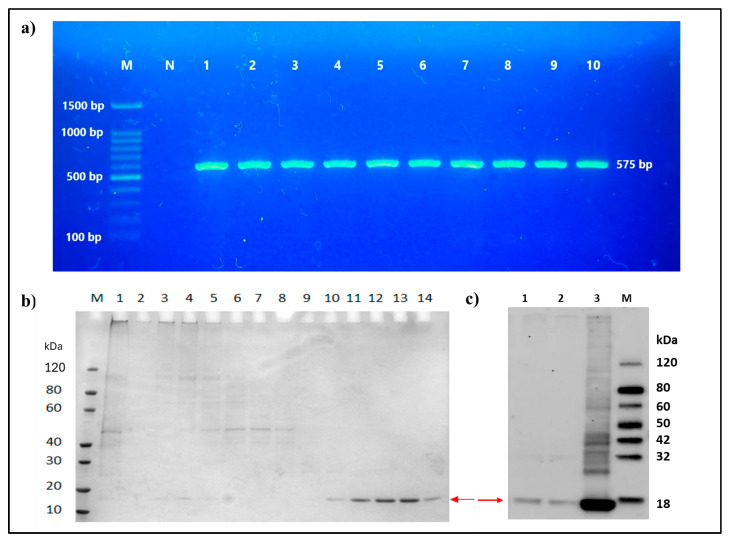
Cloning and expression of the USM.TOXOII vaccine. (**a**) PCR confirmation of the successful transformation of the pET30a.USM.TOXOII recombinant plasmid into competent *E. coli* cells. (**b**) SDS-PAGE analysis of USM.TOXOII expression; the arrow shows the eluted USM.TOXO II protein. Lane M: Protein marker. Lane 1: Loading. Lanes 2–14: Fractions of the USM.TOXOII protein. (**c**) Verification of USM.TOXOII expression by Western blotting analysis using anti-his tag antibodies; the distinct band corresponded to the expected target 17.27 kDa size. Left: Lane M: Protein marker referring to the annotated key for size. Lane 1: USM.TOXOII protein (4 h at 37 °C). Lane2: USM.TOXOII protein (16 h at 15 °C), Lane 3: Pellet of cell lysate (4 h at 37 °C).

Following the construction, USM.TOXOII gene (471 bp) was successfully cloned into the pET30a (+) expression vector. The 471 bp gene construct includes the 435 bp coding sequence for the 145 aa designed vaccine, plus the start codon, stop codon, and the NheI and HindIII restriction site sequences required for directional cloning into the vector. The pET30a.USM.TOXOII recombinant plasmid was then transformed into BL21 (DE3) pLyS competent cells, where the target antigen was expressed as a His-tagged protein (17.27 kDa), which is higher than the predicted molecular weight due to the presence of vector-derived residues, including the His-tag. The protein expression was confirmed using SDS-PAGE and Western blotting (Figure 4). The USM.TOXOII protein was purified with a Ni-NTA spin column (Qiagen, Hilden, Germany), and the purity of the purified protein was estimated to be ≥80% based on SDS-PAGE analysis under reducing conditions. The total yield of the purified protein was approximately 1.25 mg, with a concentration of 0.19 mg/mL as determined by the Bradford assay. The protein was stored at −80 °C until further use.

### 3.11. Antibody Responses in Immunized BALB/c Mice

**Figure 5:**
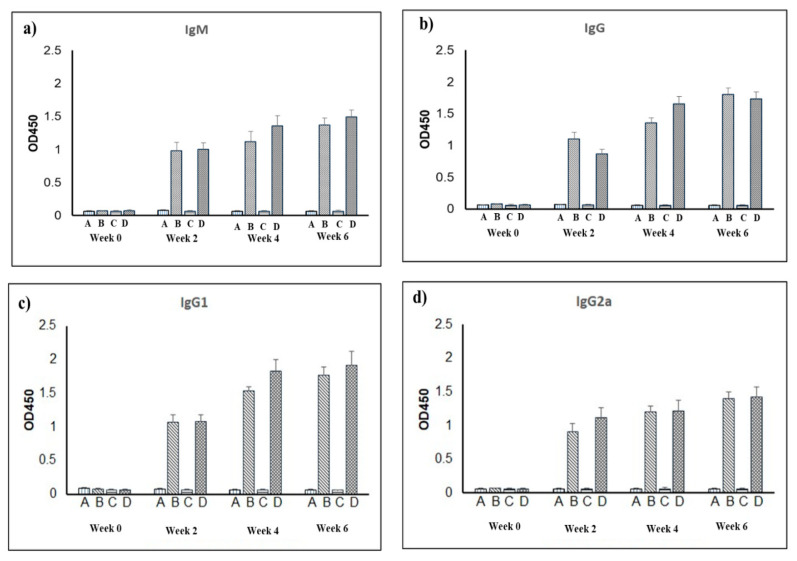
Serum levels of anti-USM.TOXOII in mice. (**a**) IgM. (**b**) total IgG, (**c**) IgG1, (**d**) IgG2a antibodies (OD_450_ ± SD) in immunized and control groups of mice, at week 0, 2, 4, and 6. A: Group A: Intraperitoneally injected with PBS (negative control). Group B: Intraperitoneally injected with USM.TOXOII. Group C: Intradermally injected with PBS (negative control). Group D: Intradermally injected with USM.TOXOII.

The measurement of USM.TOXOII-specific IgM and IgG antibodies in the serum of vaccinated mice indicated that there were elevated levels of both IgM and IgG antibody responses by the end of the immunization period (Figure 5). Nonetheless, IgM and IgG levels consistently remained low in PBS-injected animals throughout the investigation. The anti-USM.TOXOII IgM and IgG levels in inoculated mice were markedly elevated following booster vaccination, particularly at the 4th and 6th weeks post-initial immunization. Conversely, the examination of IgM and IgG synthesis in the control mice revealed no elevation in IgM and IgG titers after booster vaccinations.

The levels of IgG1, IgG2a, and IgG2b antibodies against the USM.TOXOII antigen were measured in the serum of both vaccinated and control mice. Mice inoculated with USM.TOXOII demonstrated a significant IgG1 response two weeks after the initial immunization, in comparison to those injected with PBS. The antibody response was also enhanced in the same group after the second vaccination. Following the third booster vaccination, IgG1 levels showed a consistent increase, with an average OD450 of 1.772 ± 0.1 for the intraperitoneally vaccinated group and 1.916 ± 0.2 for the intradermally vaccinated group. These values are highly significant in comparison to the control group. Conversely, the examination of the IgG2a isotype revealed a modest elevation in antigen-specific IgG2a levels, following the second and third immunizations, with mean optical density values of 1.208 ± 0.08 and 1.398 ± 0.09 in the intraperitoneally vaccinated cohorts and 1.211 ± 0.1 as well as 1.424 ± 0.1 in the intradermally vaccinated cohorts, compared to 0.060 ± 0.01 and 0.062 ± 0.007 in the control groups, respectively. Likewise, the mice vaccinated with USM.TOXOII exhibited higher IgG2b titers compared to those immunized with PBS (*p* < 0.05) two weeks post-immunization schedule completion. Moreover, a sustained elevation in IgG2b levels was seen in the vaccinated group during the second and fourth weeks in comparison to the control group (Figure S3). The control group, however, exhibited consistently low levels of IgG1, IgG2a, and IgG2b antibodies from week zero to week six when compared to the vaccinated group.

### 3.12. Cellular Immunogenicity Response

**Figure 6:**
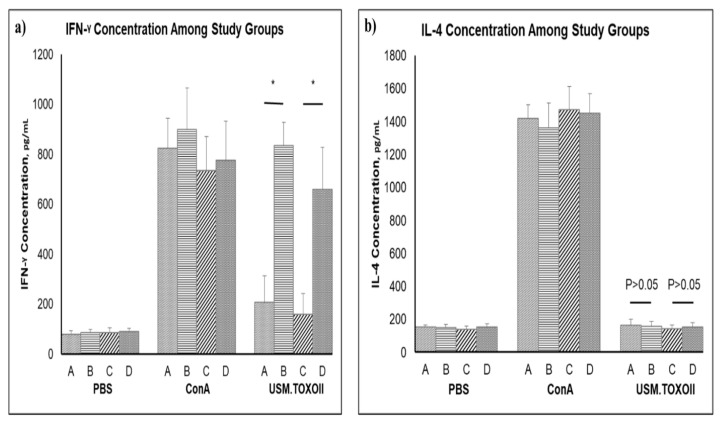
Mouse splenocytes’ in vitro production of the IFN-γ (**a**) and IL-4 (**b**) cytokines following re-stimulation with PBS, Concanavalin A (ConA), and USM.TOXOII. Group A: Intraperitoneally injected with PBS (negative control). Group B: Intraperitoneally injected with USM.TOXOII. Group C: Intradermally injected with PBS (negative control). Group D: Intradermally injected with USM.TOXOII. * *p* < 0.05 indicates a statistically significant difference compared to the PBS control group.

The induction of the Th1-specific cytokine (IFN-γ) was significantly higher in splenocytes from mice immunized with USM.TOXOII compared to those injected with PBS. The concentrations of IFN-γ reached 835.29 pg/mL for USM.TOXOII intraperitoneally immunized mice and 659.02 pg/mL for intradermally immunized mice, which were statistically significant (*p* < 0.05) compared to the IFN-γ levels (205.69 and 159.02 pg/mL) detected in the culture supernatants from the PBS-intraperitoneally and intradermally injected control mice (Figure 6).

On the other hand, the results of this study indicated that immunization with USM.TOXOII antigen did not enhance the in vitro release of IL-4 after stimulation. Likewise, no statistically significant induction of IL-4 in the PBS-stimulated splenocytes isolated from vaccinated BALB/c mice was observed, compared to control mice.

## 4. Discussion

Given the urgent need for an effective *T. gondii* vaccine and the limitations of conventional vaccine development methods, in silico approaches have emerged as a promising and efficient alternative for identifying immunoprotective antigens and designing multi-epitope vaccine candidates [13,31]. In this study, a multi-epitope antigen incorporating nine putative immunodominant epitopes from *T. gondii* was successfully developed and its immunogenic potential was evaluated. All selected CTL and HTL epitopes demonstrated 100% conservancy across multiple *T. gondii* strains, as verified using the IEDB Conservancy Analysis Tool. This ensures that the designed vaccine can elicit broad protective immunity against diverse *T. gondii* strains, rather than being limited to a single strain. The construct was successfully expressed in an *E. coli* system and purified, enabling subsequent assessment of its immunogenic properties. The purpose of creating such an epitope gene was to investigate an alternative approach for developing new vaccines to prevent *T. gondii* infections. Moreover, it was anticipated that this approach would provide good immunogenicity and immunoreactivity. Another goal of this study was to assess USM.TOXOII’s capacity to trigger a robust immune response in a mouse model.

In this study, HTL, CTL, and LBL epitopes were successfully joined using GPGPG, AAY, and KK linkers to create the multi-epitope vaccine. Given that they improve the vaccine’s folding, expression, and stability, linkers are crucial in vaccine construction [32]. Since the GPGPG linker inhibits the production of junctional epitopes and promotes immunological processing, it was utilized to conjugate the CTL epitopes [33]. Moreover, because AAY linkers improve epitope presentation, decrease immunogenicity, and alter protein stability by showing the proteasomal cleavage site, they were utilized to conjugate the HTL epitopes [34]. Additionally, the LBL epitopes were conjugated using KK (bi-lysine) linkers, which preserve the epitopes’ autonomous immunogenic properties [13].

Given that antigens with molecular weights of more than 5–10 KDa are considered strong immunogens, the vaccine construct’s average molecular weight of 14.8 kDa qualifies it as a good immunogenic protein [35]. Moreover, with a theoretical PI of 8.69, the vaccine was shown to be basic and might need to be optimized to avoid resistance of *T. gondii*. Solubility is another crucial aspect of a vaccine, and the vaccine construct’s solubility was determined to be 0.515555, indicating that it is soluble, which is advantageous for obtaining a high protein output. The vaccine also appeared to be thermostable throughout a broad temperature range, as indicated by its aliphatic index of 76.76 (>70). A positive GRAVY test further indicates that the vaccine is hydrophobic and less soluble in water. This can be advantageous for vaccines because hydrophobic molecules tend to have better stability, making them less prone to degradation under environmental stresses. This stability can additionally enhance the vaccine’s shelf life and its overall effectiveness during storage and transport. In addition, hydrophobic properties can promote better interaction with lipid membranes, aiding in antigen delivery and presentation to the immune system. These characteristics are particularly beneficial when combined with appropriate formulation strategies to ensure optimal solubility and bioavailability for administration.

The secondary structure of a polypeptide chain is represented by the hydrogen bonding scheme between the carboxyl oxygen atoms and amino hydrogen, which mostly consists of α-helices and β-structures. The high hydrogen-bond strength of the alpha-helix and beta-turn inside the protein structure additionally acts to maintain the protein’s shape, which results in a positive interaction with antibodies [36,37]. As predicted by PSIPRED server’s analysis of the vaccine’s secondary structure, there were 37.9% α helices, 12.4% β-strands, and 49.7% random coils. The vaccine’s high proportion of α helices and β-strands guarantees high-energy hydrogen bonding, which preserves the protein’s structure and potent antibody engagement. However, complexes of MHC and T-cell receptors rely on the spatial organization of proteins, and immunogenicity cannot be predicted just by a linear sequence. Interestingly, given that the vaccine had superior 3D quality, as demonstrated by the trRosetta server, the model with a higher TM score was chosen for 3D structure prediction [38].

Galaxy refinement aids in the reconstruction and repackaging of the side chain in the 3D model, and thus significantly improves the overall quality of the molecular dynamic simulation [39]. A number of quality metrics, including GDT-HA (0.9931), RMSD (0.282), MolProbity (2.231), the absence of rotamers, and Rama preferred (94.4), were taken into consideration while choosing the improved model 1. GDT-HA determines the protein model’s overall quality, whereas RMSD focuses on the differences in angles and bond lengths between the crude and refined models. Consequently, the better the revised model is compared to the original structure, the lower the RMSD. Additionally, a model having a lower MolProbity score over the average structure at the crystallographic resolution is of higher quality [40]. Resultantly, 97% of the amino acid residues were in the preferred area in PROCHECK SERVER, according to the validation of the 3D structure using Ramachandran plot analysis. The ERRAT tool projected that the structure will have a total quality score of 96.3235, higher than the commonly accepted value of >50% for high-quality models [41]. According to the Q mean value of −3.58, the refined vaccine’s quality was also within the boundaries of a non-redundant set of PDB structures necessary for a protein with acceptable quality. Moreover, VERIFY 3D revealed that 57.24% of the amino acids had an average 3D-1D score > 0.1.

Recognition of any vaccine constructs by host immune receptors is a key determinant of immunogenicity in reverse vaccinology. Among these receptors, TLR4 plays a critical role in initiating innate immune responses and promoting Th1-polarized immunity through cytokines such as IL-12 and IFN-γ, which are essential for controlling *T. gondii* infection. Therefore, the interaction between TLR4 and USM.TOXOII was evaluated using molecular docking. The results showed a docking score of −1039.6, suggesting a strong predicted interaction between the vaccine construct and TLR4. However, this docking result should be interpreted only as an exploratory in silico prediction of a possible interaction under the computational conditions employed. The docking score alone does not establish biologically meaningful receptor binding, receptor activation, engagement of innate immune pathways, or a contribution to the immune responses observed in this study. Therefore, the stability and functionality of the USM.TOXOII–TLR4 complex under physiological conditions, as well as any role of TLR4 signaling in the observed IFN-γ response, would require direct experimental validation.

The potential of the USM.TOXOII antigen to trigger an immune response was confirmed in BALB/c mice. The total IgG and IgG subclass (IgG1, IgG2a, and IgG2b) levels, the IgM, IL-4, and IFN-γ were evaluated. The IgG1 and IL-4 are often associated with Th2, while IgG2a and IFN-γ are associated with the Th1-type immune response [42]. The better immunogenicity of the intradermal method was demonstrated by this study, which showed that at all time points, intradermal immunization produced noticeably higher IgM levels than intraperitoneal vaccination. The abundance of antigen-presenting cells in the skin, which promoted effective immune activation, was probably the cause of this response. Additionally, the specificity of the vaccine-induced immune response was confirmed by the consistently low IgM levels in both control groups. These results prove that intradermal vaccination may promote significant humoral immunity due to its significant antibody production, consistent with studies showing its benefits over systemic delivery techniques like intraperitoneal injection [43].

Conversely, IgG antibodies have been shown in several trials to significantly prevent *T. gondii* infection [44]. Indeed, phagocytosis, complement system activation, and parasite receptor blockage are some ways in which parasite death is made possible by inducing the formation of anti-*T. gondii* IgG antibodies. Protection against toxoplasmosis has been found to be directly associated with these mechanisms [45]. In this study, the sera of mice inoculated with USM.TOXOII exhibited noticeably higher levels of total IgG antibodies than those of animals injected with PBS. It should be noted that the serological analyses in this study were performed using pooled sera from each experimental group rather than on serum samples obtained from individual mice. While this approach provides a reliable estimate of the group-level humoral immune response and is consistent with previous immunogenicity studies [46,47], it does not capture inter-individual variability among mice within the same experimental group. Indeed, individual serum analysis would allow assessment of response consistency across the vaccinated mice. Moreover, there was a minor but gradual difference in the level of the USM.TOXOII-specific IgG antibodies in the sera from intraperitoneally and intradermally vaccinated mice with booster immunization, particularly in the 4th and 6th weeks following the first immunization. Mice vaccinated with USM.TOXOII showed increased IgG1 levels at weeks 4 and 6 compared to their respective control groups, while both vaccinated groups exhibited a moderate increase in anti-USM.TOXOII IgG2a levels following the second and third immunizations. In agreement with this study’s findings, Eichinger et al. (2020) previously noted that antigen-specific IgG1 and IgG2a antibody subclasses could be elicited by developing protective immunity [48]. Similarly, numerous studies have shown that mice immunized with recombinant antigens develop both IgG1 and IgG2a antibody responses, reflecting a broad humoral immune response [14]. On the other hand, it is often stated that the Th1 immune response predominates throughout the assessment of many *T. gondii* vaccines [49].

The result of the current study revealed that the inoculated mice had a considerably higher level of IFN-γ than the control animals. IFN-γ levels were also marginally greater in the intraperitoneal group than in the intradermal group. Nevertheless, following splenocyte stimulation, vaccination with the USM.TOXOII antigen did not enhance the in vitro release of IL-4. These findings imply that USM.TOXOII-induced cellular immunity is a Th1-type response. Similarly, cellular immunity was shown to differ somewhat from humoral immunity. IgG1 antibody isotype predominated in the combined Th1- and Th2-type immune response triggered by USM.TOXOII. Meanwhile, the cytokine analysis showed a robust Th1 immune response after in vitro stimulation. Similar results have been documented previously. BALB/c mice immunized with a multi-epitope DNA vaccine developed protective immunity, IgG1 and IgG2a antibody responses, and in vitro generation of IFN-γ cytokines [17]. According to another study, high levels of toxoplasma-specific IgG2a, IgG1 and IFN-γ indicated efficient humoral and cell-mediated immune responses following TLA vaccination [10]. The produced immunity considerably decreased the number of brain cysts in vaccinated mice. Given that TLA is a combination of many *T. gondii* antigens, the protective immunity that inoculated mice experience is most likely the result of TLA’s capacity to trigger both innate and adaptive immune responses. Overall, USM.TOXOII demonstrated promising immunogenic properties and warrants further investigation as a potential vaccine candidate against *T. gondii*. However, its protective efficacy remains to be validated through challenge studies. In addition, cytokine analysis was limited to IFN-γ and IL-4 measurements and did not include other key cytokines associated with Th1/Th2 responses, such as IL-2, TNF-α, and IL-10. Furthermore, endotoxin testing of the purified recombinant protein was not performed, and therefore a potential contribution of residual *E. coli*-derived endotoxin to the observed IFN-γ response cannot be entirely excluded. Further studies incorporating a broader assessment of immune responses and protection against infection are warranted. It should also be noted that epitope prioritization was optimized for human HLA presentation, and murine MHC compatibility was not formally evaluated during the computational design phase; therefore, the BALB/c mouse experiment serves as an exploratory preliminary immunogenicity model only.

## 5. Conclusions

In conclusion, the present study provides preliminary evidence that USM.TOXOII possesses immunogenic potential and is capable of inducing both humoral and cellular immune responses in BALB/c mice. The USM.TOXOII multi-epitope vaccine incorporating immunogenic T- and B-cell epitopes within the *T. gondii* GRA14, GRA1, and SAG1 could be a suitable model for further investigations to obtain an effective vaccine against toxoplasmosis. However, challenge trials with live *T. gondii* tachyzoites are necessary to validate the complete protection of the developed vaccine in the immunized mice.

## Figures and Tables

**Table 1:** The physiochemical and antigenic properties of USM.TOXOII vaccine construct.

Characteristics	Values	Remarks
The quantity of amino acids	145	Suitable
Molecular weight	14,837.81	Medium
Theoretical PI	8.69	Basic
Formula for a chemical	C_664_H_1027_N_175_O_201_S_5_	-
Extinction coefficient (at 280 nm in H20)	21,680	-
Estimated half-life (mammalian reticulocytes, in vitro)	1.1 h	-
Estimated half-life (in vivo, yeast)	3 min	-
Estimated half-life (*E. coli*, in vivo)	2 min	-
Vaccine instability index	34.85	-
Aliphatic index of vaccine	76.76	Stable
Grand average of hydropathicity (GRAVY)	0.163	Hydrophobic
Antigenicity	0.7758	Antigen
Solubility	0.515555	Soluble

## Data Availability

The data generated in this study are available within the manuscript and Supplementary Materials.

## Supplementary Materials

The following supporting information can be downloaded at: https://www.mdpi.com/article/10.3390/vaccines14060545/s1, Data S1: Nucleotide sequence of USM.TOXOII gene open reading frame (ORF); Figure S1: Predicted chains of the vaccine’s secondary structure by PSIPRED; Figure S2: Molecular docking of the USM.TOXOII vaccine construct with TLR-4; Figure S3: Serum levels of anti-USM.TOXOII IgG2b antibody; Figure S4: Unprocessed images of Figure 4; Table S1: Antigenic prediction scores of the three representative *Toxoplasma gondii* proteins; Table S2: The top LBL epitopes that selected for vaccine construction; Table S3: Selected CTL epitopes that fulfilled all inclusion criteria for final vaccine construction; Table S4: HTL epitopes selected for vaccine construction.
