# Supplementary material for: Immunogenicity of a Recombinant Multi-Epitope Vaccine Incorporating GRA14, SAG1, and GRA1 Antigens of Toxoplasma gondii in BALB/c Mice
Source: Vaccines (Basel). 2026 Jun 20;14(6):545. doi: 10.3390/vaccines14060545 (PMC13307803; doi:10.3390/vaccines14060545)
Supplement: Supplementary file 1 [file vaccines-14-00545-s001.zip › vaccines-4349123-supplementary.pdf]

Immunogenicity of a Recombinant Multi-Epitope Vaccine Incorporating GRA14, SAG1, and GRA1 Antigens of *Toxoplasma gondii* in BALB/c Mice

Data S1 Nucleotide sequence of USM.TOXOII gene open reading frame (ORF)

>USM.TOXOII_ORF

ATGTTTTCACCCTTCCTATTATATTTGCAAGACGCCGTAGGCGAGTTCGGCCCAGGTC
CGGGGGTGACCTGCCCGGATAAGAAGTCCACCGCAGCGGTGATCCTGACCGGTCCG
GGTCCGGGCGTTCGTGTTAGCGCCATTGTGGGCGCGGCGGCTTCGGTGTTCGTGGCG
GCTTACATCATGGAAGATCATGTTGCGAGCGTCGCGGCTTACCAGACCTTTGTTGTT
GGCTGCATTAAAGCAGCGTACGCAATCGTGGGCGCGGCGGCTTCCGTCAAAAAGCG
CGGTGACCGTAGCAGTGGCTGGTCATCTTGTAGCTGGTTGTTTTATAAAAAGGTTCC
GCAGGATAACAACCAATATTGCAGCGGTACTACGCTGACCGGTAAAAAGGGTGCGT
ATGCCGCGGAGGGTGGTGACAATCAAAGCTCTGCAGTTAGCCATCATCACCACCAC
CACTAA

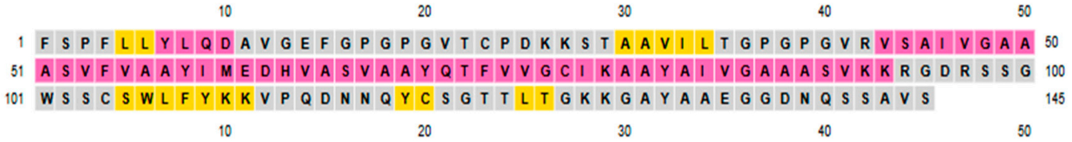

Figure S1 Predicted chains of the vaccine’s secondary structure by PSIPRED

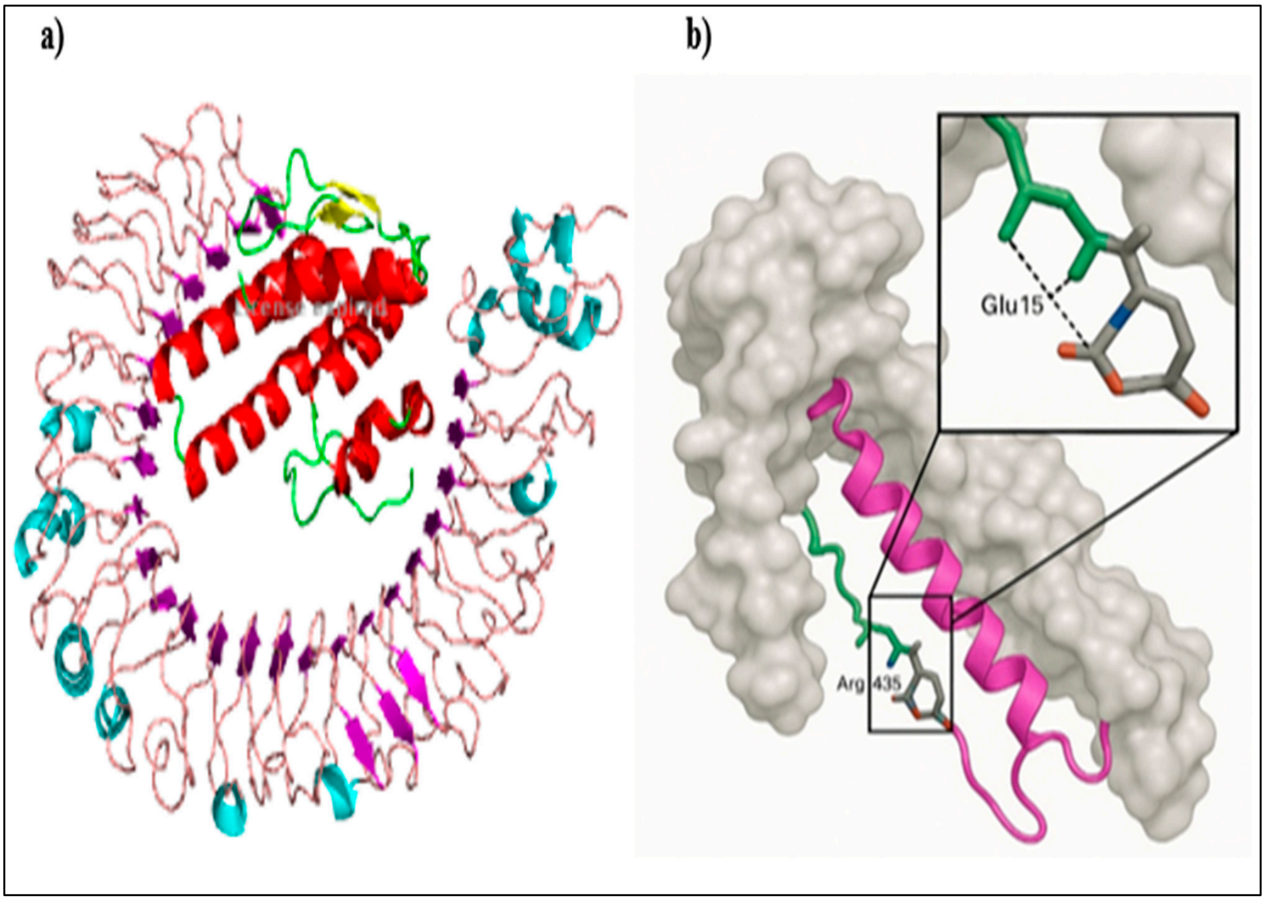

Figure S2 a) Molecular docking of the USM.TOXOII vaccine construct with TLR-4 using Cluspro. b) Molecular docking visualisation highlighting key molecular interactions at the binding interface (Binding affinity:-1039.6 Kcal/mol).

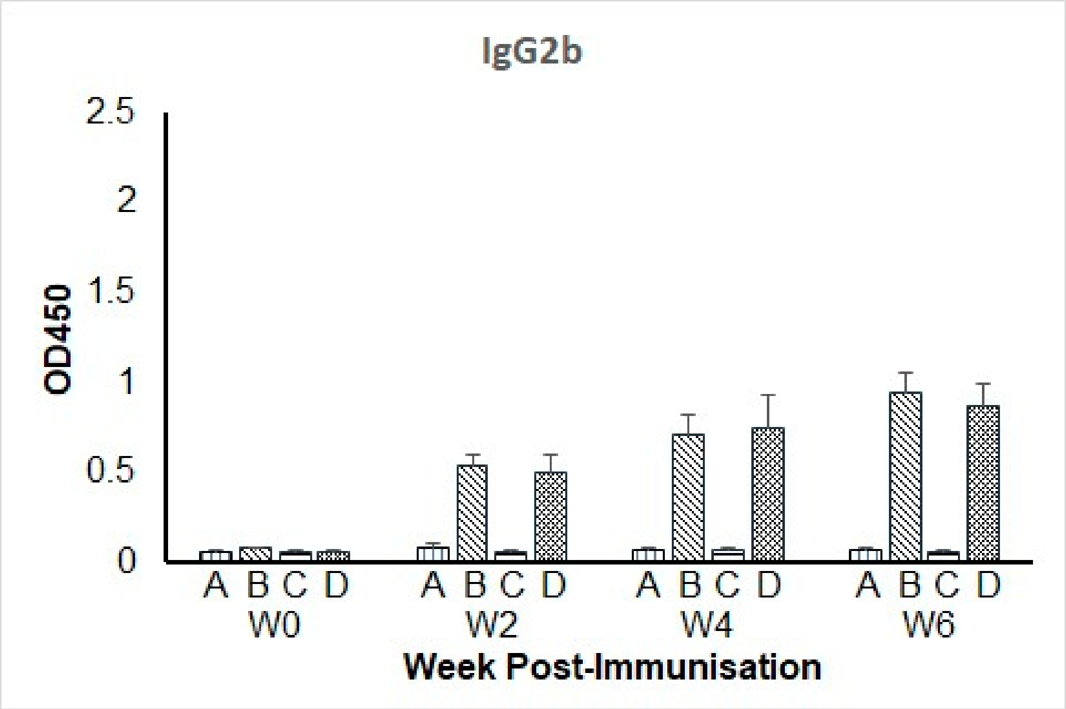

Figure S3 Serum levels of anti-USM.TOXOII IgG2b antibody ($OD_{450} \pm$ SD) in immunized and control groups of mice, at week 0, 2, 4, and 6. A: Intraperitoneally injected with PBS. B: Intraperitoneally injected with USM.TOXOII. C: Intradermally injected with PBS. D: Intradermally injected with USM.TOXOII

Table S1 Antigenic prediction scores of the three representative *T.gondii* proteins

Antigenicity (Threshold=0.4)				
**Protein name**	**Accessionnumber**	**Length**	**Value**	**Remark**
GRA14	AXG50779.1	408	0.5337	Antigenic
SGA1	AAO61460.1	319	0.7719	Antigenic
GRA1	KAF4642298.1	190	0.4815	Antigenic

Table S2 The top three LBL epitopes that selected for vaccine construction

Protein	LBL epitopes	LBC epitope	Antigenicity	Allergenicity	Toxicity	Human homology
**GRA14**	GRA14_A	RGDRSSGWSSCSWLFY	1.3632	No	No	Non-homology
**SAG1**	SAG1_A	VPQDNNQYCSGTTLTG	1.1219	No	No	Non-homology
**GRA1**	GRA1_A	GAYAAEGGDNQSSAVS	1.2907	No	No	Non-homology

Table S3 The selected CTL epitopes that fulfilled all inclusion criteria for final vaccine construction

Protein	Name	CTL epitopes	C-score	Immuno genicity	Antigenicity (threshold:0.4)	Allergenicity	Toxicity	Conservancy	Human homology
GRA14	GRA14_B	IMEDHVASV	1.2275	0.6239	0.06154	Non-Allergen	Non-Toxin	Conserved	Non-homology
SAG1	SAG1_B	QTFVVGCIK	0.9869	1.3264	0.1839	Non-Allergen	Non-Toxin	Conserved	Non-homology
GRA1	GRA1_B	AIVGAAASV	0.8959	0.8860	0.05879	Non-Allergen	Non-Toxin	Conserved	Non-homology

Table S4 The HLT epitopes selected for vaccine construction

Protein	Protein	HTL epitope	Antigenicity	IFN	IL4	IL10	Allergenicity	Toxicity	Conservancy	Human homology
GRA14	GRA14_C	FSPFLLYLQDAVGEF	0.5264	Induced	Non-IL4-inducer	IL10 inducer	Non-Allergen	Non-Toxin	Conserved	Non-homology
SAG1	SAG1_C	VTCPDKKSTAAVILT	0.8626	Induced	Non-IL4-inducer	IL10 inducer	Non-Allergen	Non-Toxin	Conserved	Non-homology
GRA1	GRA1_C	VRVSAIVGAAASVFV	1.0946	Positive	Non-IL4-inducer	IL10 inducer	Non-Allergen	Non-Toxin	Conserved	Non-homology

Figure S4. Unprocessed images of Figure 4

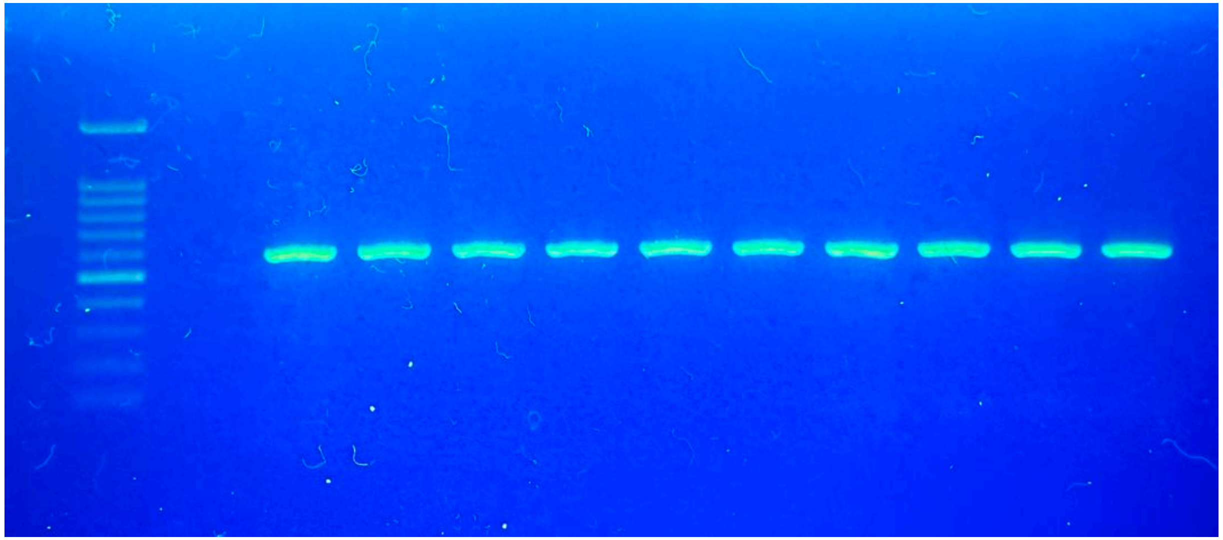

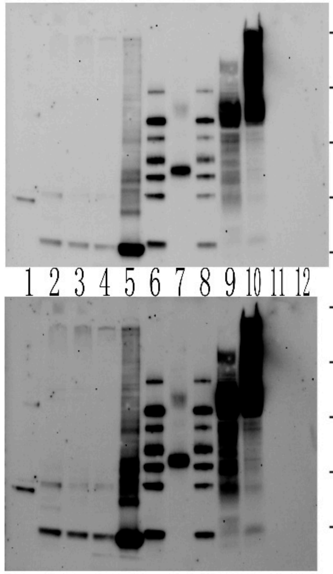

1				
2	38186K, U799TIC210-1, 15.791		20	His
3	38186K, U799TIC210-1, 15.791		20	His
4	38186K, U799TIC210-1, 15.791		10	His
5	38186K, U799TIC210-1, 15.791		10	His
6	M	N/A	6.5	His
7				
8	M	N/A	3	His
9				
10				
11				
12				
